# Non-steroidal anti-inflammatory drug induced acute kidney injury in the community dwelling general population and people with chronic kidney disease: systematic review and meta-analysis

**DOI:** 10.1186/s12882-017-0673-8

**Published:** 2017-08-01

**Authors:** Xinyu Zhang, Peter T Donnan, Samira Bell, Bruce Guthrie

**Affiliations:** 1 Division of Population Health Sciences, University of Dundee, The Mackenzie Building, Kirsty Semple Way, Dundee, DD2 4BF UK; 2 Renal Unit, Ninewells Hospital, Dundee, UK

**Keywords:** Acute kidney injury, Chronic kidney disease, Non-steroidal anti-inflammatory drugs, Pharmacoepidemiology

## Abstract

**Background:**

Non-steroidal anti-inflammatory drugs (NSAIDs) are a common cause of adverse drug events (ADEs), but renal risks of NSAIDs are less well quantified than gastrointestinal and cardiac risks. This paper reports a systematic review of published population-based observational studies examining the risk of acute kidney injury (AKI) associated with NSAIDs in community-dwelling adults and those with pre-existing chronic kidney disease (CKD).

**Methods:**

MEDLINE and EMBASE databases were searched until June 2016, and 3789 papers screened. Ten studies reporting NSAID risk of AKI in the general population were included in random effects meta-analysis, of which five additionally reported NSAID risk in people with CKD.

**Results:**

In the general population, the pooled odds ratio (OR) of AKI for current NSAID exposure was 1.73 (95%CI 1.44 to 2.07), with somewhat higher risk observed in older people (OR 2.51, 95%CI 1.52 to 2.68). In people with CKD, individual study OR of AKI due to current NSAID exposure ranged from 1.12 to 5.25, with pooled estimate OR 1.63 (95% CI 1.22 to 2.19).

**Conclusions:**

No study reported baseline risk of AKI in different populations meaning absolute risks could not be estimated, but baseline risk and therefore the absolute risk of NSAID exposure is likely to be higher in people with CKD and older people. Large population based studies measuring AKI using current definitions and estimating the absolute risk of harm are needed in order to better inform clinical decision making.

**Electronic supplementary material:**

The online version of this article (doi:10.1186/s12882-017-0673-8) contains supplementary material, which is available to authorized users.

## Background

Non-steroidal anti-inflammatory drugs (NSAIDs) are commonly prescribed in primary care for their analgesic, antipyretic and anti-inflammatory effects. One in fifteen US adults are actively prescribed NSAIDs at any one time [1], and in many countries low-dose preparations are also available over-the-counter (OCT). Partly due to their widespread use, NSAIDs account for 25% of adverse drug events (ADEs) reported in the United Kingdom ﻿﻿(UK) and 21% in the United States (US) [2, 3]. NSAIDs are also commonly implicated in hospital admissions due to ADEs, including those which are fatal [4], but gastrointestinal and cardiac toxicity are better quantified than renal toxicity [5].

NSAIDs can reduce renal blood flow, cause tubular obstruction through crystal deposition, and induce direct cytotoxicity and cell-mediated immune injury mechanisms leading to the occurrence of acute kidney injury (AKI). Another symptom that is commonly caused by NSAIDs is interstitial nephritis (AIN) which requires specialist review, renal biopsy, high-dose corticosteroid and/or immunosuppressant treatments, and will normally be progression in chronic kidney disease (CKD) [6]. Older age [7, 8] and underlying chronic kidney disease are also related to the onset of AKI during NSAID use, with early studies showing that the risk of deterioration in renal function increases 3–4 fold in patients with abnormal baseline renal function compared to those with normal renal function [9]. Notably, NSAIDs are commonly prescribed to people with CKD, despite guidance to avoid them in this population. In US veterans in 2005, 15.4% of people with CKD were prescribed traditional NSAIDs or COX-2 inhibitors [10], compared to 11.1% of people with CKD in the UK in 2012 [11], and 15.9% of people with CKD in Australia in 2004–2006 [12]. Better quantification of risk in people with CKD is therefore of particular clinical interest, as is whether NSAID risk varies by age and by COX-2 selectivity. In terms of COX-2 selectivity, early studies suggested that COX-2-selective inhibitors caused fewer renal adverse effects including reduction in glomerular filtration rate (GFR), increased serum creatinine (SCr) and hypertension [13–15]. Other studies have shown no significant differences in renal risk between COX-2-selective inhibitors and nonselective NSAIDs [16, 17].

There is little evidence about the risk of AKI associated with NSAID use in people with CKD available from randomised trials of NSAIDs because such trials routinely exclude people with CKD and rarely report renal outcomes [18]. Under these circumstances, observational evidence provides the best guide to practice that exists. The aim of this study is to systematically review published high-quality population-based observational studies to quantify the risk of AKI due to NSAIDs in the general population and in people with pre-existing CKD.

## Methods

### Data sources and search strategy

MEDLINE and EMBASE were systematically searched from inception to June 21th 2016 using OVID from the Knowledge Network using a predetermined list of keywords including NSAIDs, renal diseases and renal function measurements modified from the search strategies used by two related Cochrane reviews (see Additional file 1 for search strategy) [18, 19]. Search results were restricted to cross-sectional, cohort and case-control studies in the English language. The reference lists from all identified primary studies, review articles, Kidney Disease Improving Global Outcomes (KDIGO) clinical practice guidelines for CKD and AKI and OpenSIGLE (unpublished literature database) were manually checked to screen for additional relevant papers.

Citations were independently screened for eligibility by two reviewers based on title and abstract (XZ and SB or XZ and BG). If one or more authors deemed the study potentially relevant, or if there was any uncertainty about eligibility based on title and abstract alone, then the full text paper was retrieved for review. Authors of original studies were not contacted. Study selection and quality assessment from full-text papers retrieved were performed independently by two reviewers (XZ and BG).

### Study selection

The search strategy and data extraction were defined in a PICOS format (participants, intervention, comparison, outcome and study design). Studies published in English were eligible for inclusion when they used observational methods to study adults in the community exposed to NSAIDs and reported AKI as an outcome. Given the historical lack of consensus on AKI definition, studies using a variety of definitions of AKI were included, with AKI defined by International Classification of Diseases (ICD) 9 or 10 codes, or change in eGFR or creatinine clearance (CrCl) or serum creatinine (SCr). Similarly, varying definitions of CKD were allowed including estimated GFR < 60 ml/min (with or without standardization to body surface area), or based on ICD codes, or SCr > 122 μmol/L, or structured patient interview. Traditional NSAIDs and COX-2 inhibitors were included with the exception of low dose aspirin (<300 mg per dose) [20]. Studies were excluded if they were published in abstract only, included children (age < 18 years old), only included post-operative patients or others receiving only one or two doses of NSAID as treatment (e.g. for renal colic or post-lithotripsy), or had end-stage renal disease defined as being on dialysis or having received a renal transplant. Finally, meta-analyses, studies with <100 subjects, and studies without a contemporaneous control group drawn from the same population were excluded.

### Data extraction and quality/validity assessment

Data were extracted into a standardised form and checked for accuracy by a second reviewer. When data were reported in strata, the data were extracted as separate subgroups. The following data were extracted for each included study: author, publication year, study design, population (data source, sample size, location, age, gender and underlying renal conditions), definition of AKI, inclusion criteria, exclusion criteria, medication exposure (type of NSAID), period and length of NSAID usage, number of people who were and were not exposed to NSAIDs, as well as crude unadjusted and adjusted associations between NSAID use and outcomes. The quality of the included studies was evaluated in three domains using the validated Newcastle-Ottawa Quality Assessment Scale for cohort and case-control studies [21], with each item rated as either one star or missing (Table 1). Disagreements were resolved by discussions with two authors (XZ and BG) and a third reviewer was involved where required (PTD).

**Table 1:**
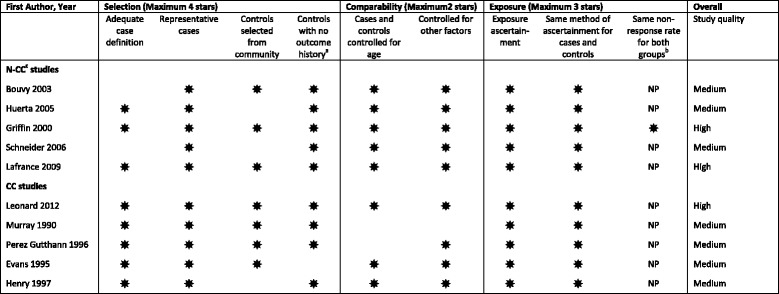
Quality assessment

^a^If cases are first occurrence of outcome, then it must explicitly state that controls have no history of this outcome. If cases have new (not necessarily first) occurrence of outcome, then controls with previous occurrences of outcome of interest will not be excluded

^b^ = same non-response rate for both groups reported ; NP = not reporting non-response rate is Not a Problem (since bias is less likely if the cases and controls come from the same population and have outcomes and exposures ascertained in the same way)

^c^ *CC* case-control, *N-CC* nested case-control

### Statistical analyses

The outcome was the presence or not of AKI. In studies among the general population, adjusted odds ratio (OR) with 95% confidence intervals (CI) for AKI with NSAID exposure were pooled using the generic inverse variance method which assumed weights equivalent to inverse of variance of individual estimates [22, 23]. This was because adjusted ORs and CIs were mostly presented in the primary studies while raw ORs and CIs were not. Moreover, pooled results will be more meaningful with the adjustment. When calculating the pooled result, in order to be more conservative, the individual OR with the most extreme of the lower or higher side of the CI for each study was used to estimate the variance. Additional subgroup meta-analyses were conducted to explore heterogeneity stratified according to pre-specified study-level covariates namely age and COX-2 selectivity.

In all but one case, primary studies which included analysis for the subgroup of people with CKD did not provide adjusted estimates of association. Therefore crude ORs and CIs from the raw data were calculated, and pooled using Mantel-Haenszel method.

A random-effects model was used for all analyses, and heterogeneity between studies assessed by the I^2^ statistic and the χ^2^ test for heterogeneity. I^2^ is the percentage of variance that is due to between-study variance and is an indicator of consistency between studies. Values of 25–50%, 50–75% and >75% were considered evidence of mild, moderate and marked heterogeneity, respectively [24]. Publication bias was not assessed because of the extensive statistical heterogeneity found, since such heterogeneity in itself may lead to funnel plot asymmetry [25]. AKI is an uncommon adverse event and it was assumed that the OR was an accurate estimate of the relative risk (RR) of AKI in NSAID users compared with non-users.

Statistical analyses were performed using Review Manager 5.2 (Cochrane Collaboration, Oxford, United Kingdom). Statistical significance was set at *P* < 0.05 for all analyses. This systematic review was structured in accordance with the Meta-analysis of observational studies in epidemiology (MOOSE) statement (Additional file 2) [26].

## Results

### Study flow and characteristics

**Fig. 1:**
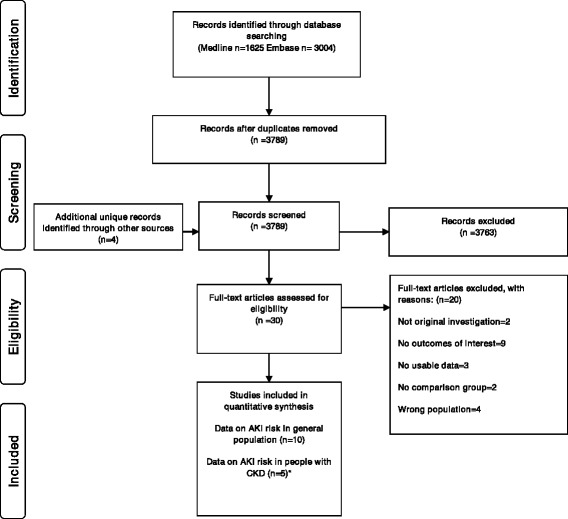
Flow diagram of the identification process for eligible studies

Electronic searches retrieved 4629 citations, with 3789 unique citations screened and four studies [27–30] identified from other sources (Fig. 1). After title and abstract screening 30 full-text studies were reviewed of which 10 studies published between 1990 and 2012 met the inclusion and quality criteria and were included (details for excluding were recorded in Additional file 3). All 10 studies examined NSAID-associated AKI risk in the general population with a total of 1,609,163 participants [6, 8, 9, 31–37]. Five of these studies also provided data in the subset of people with CKD [31–33, 35, 37]. Eighty percent of studies were rated with seven or more stars out of a possible nine on the Newcastle-Ottawa quality assessment scale (Table 1). Hence the quality of the included studies was considered to be medium to high.

In all included studies, eligible cases with AKI were recruited in a defined catchment area over a defined period of time, the ascertainment of exposure was through secure electronic records and the same method of ascertainment was used among cases and controls. Eighty percent of the included studies had independent validation of cases while the remaining 20% relied on record linkage alone (ICD codes in database) with no reference to the primary record. One study [33] used hospital controls as their cases were people with community exposure to NSAIDs admitted to hospital with AKI. Two studies [34, 37] used a mixture of hospitalized and community controls. The other included studies selected controls from community or reported hospital and community controls separately in which case only comparisons with community controls were included. The history of outcome occurrence in cases and controls were adequate in 90% of the studies. Two included studies did not match for age and other factors among cases and controls while one of them adjusted for a list of confounders including sex and use of prescription of acetylsalicylic acid (ASA) in current use of any NSAID.

Table 2 summarises the 10 included nested case-control and case-control studies (five each), of which five involved participants from North America, four from Europe and one from Australia. Nine studies used data extracted from routine electronic databases while the Australian study [33] combined electronic data with structured patient interviews. One study population comprised patients taking angiotensin-converting-enzyme inhibitor (ACE inhibitors) and the others were of the general adult population [8]. Seventy percent of the studies focused on older participants (either only including older participants or where the mean/ median age of participants was >65 years) while gender proportions among included studies were diverse. Current or new use of NSAIDs as reported by the original study authors was used as the exposure definition (70% of studies reported exposure to NSAIDs 0–90 days prior to the index day for the adverse event, other studies did not specify). One study only examined ibuprofen [9], but most examined exposure to a variety of NSAIDs. Half of the studies used laboratory data to define the presence of AKI, whereas varying sets of hospitalisation discharge diagnosis ICD codes were used in the other half. Each of the studies adjusted for a list of confounders. Newer studies tended to have a more thorough adjustment. The most common confounders studies adjusted for general polulation are age, gender, comorbidity (such as hypertension, diabetes, heart failure, and cardiovascular disease), concomitant drug use (such as diuretics, antibiotics, radio contrast exposure, and nephrotoxic drugs), and hospitalization (Table 3).

**Table 2:** Characteristics of included studies^a^

First Author, Year	Design; Data; Country	Inclusion Criteria	Exclusion Criteria	Participants	Mean Age (yr)	% Male	Definition of NSAID use (exposure)^b^	Definition of AKI (outcome)	Adjusted OR (95%CI) for general population, plus crude OR for CKD pop if available
Bouvy 2003 [8]	N-CC; PHARMO record linkage system; The Netherlands	>40 years, with ≥2 consecutive prescription for an ACEI	Hospitalisation with renal problems before start an ACEI	144 cases and 1189 controls	Not reported (all >40 years)	63.9 cases; 45.4 controls	New/ start of ≥1 prescription in 3 months before hospital admission	Hospitalisation ICD9 584 or 586	2.20 (1.10,4.50)
Huerta 2005 [34]	N-CC; GPRD; UK	50–84 years, ≥2 years enrolment with GP and ≥1 year since first computerized prescription	Cancer, renal disorder, cirrhosis, systemic connective tissue disease	386,916 individuals	Not reported (all >50 years)	Not reported	Supply for the most recent prescription lasted until 0–30 days before index date	Clinical diagnosis by a specialist, and SCr >1.7 mg/dl (150 μmol/L) or urea level >47.6 mg/dL (17.0 mmol/L)	3.23 (1.79,5.82)
Leonard 2012 [6]	CC; GPRD; UK	Not reported	History of kidney transplant, having outcome of interest during baseline period	27,982 cases and 1,323,850 controls	68.6 cases; 66.9 controls^c^	49.7 cases; 50.4 controls	Active orally administered tNSAID therapy	Diagnostic codes described in succeeding texts supplemented by GP’s free-text	1.31 (1.25,1.37)
Murray 1990 [9]	CC; Regenstrief Health Center; US	>18 years, received ibuprofen or acetaminophen during 11May1975- 29Sept1986, baseline and post-prescription SCr and BUN results available	Prescriptions of other NSAIDs, SCr < 0.3 mg/dL (30 μmol/L), BUN < 5 mg/dL (1.8 μmol/L)	4790 cases and 8205 controls	Not reported	27.4 cases N/A controls	Patients received first prescription of ibuprofen between 11May1975- 29Sept1986	Patients with normal baseline values, SCr >1.2 mg/dL (110 μmol/L) or BUN > 18 mg/dL (6.4 μmol/L); Patients with elevated baseline values, SCr or BUN ≥10%increase	1.05 (0.88,1.26)
Perez Gutthann 1996 [36]	CC; Saskatchewan health plan information; Canada	≥1 NSAID prescription during study period	Malignant neoplasm, CRF, in-hospital disease onset, insufficient data, other systemic/ renal conditions	228,392 members	Not reported	45.5	Most recent prescription filled 0–30 days before index	ICD9 580.9, 581, 583.2, 583.6–583.9, 584, 586, 593.9	4.10 (1.50,10.8)
First Author, Year	Design; Data; Country	Inclusion Criteria	Exclusion Criteria	Study Sample	Mean Age (yr)	% Male	Definition of NSAID use (exposure)^b^	Definition of AKI (outcome)	Adjusted OR (95%CI) for general population, plus crude OR for CKD pop if available
Evans 1995 [31]	CC;MEMO; UK	Resident in Tayside, Scotland registered with a Tayside GP in May 1990	Not reported	320 patients and 1238 community controls^d^	Not reported	Not reported	≥1 oral NSAID prescriptions dispensed during 90 day period prior to the index date	ICD9 583.8, 584.5, 584.7–584.9	2.20 (1.49,3.25); Crude OR for CKD population 3.04 (1.68,5.49)
Griffin 2000 [32]	N-CC; Tennessee Medicaid enrolment files; US	≥65 years, enrolled in Medicaid ≥ 1 year	End-stage renal disease, hospital-acquired acute renal failure, incomplete demographic data, remote counties residents	7145 patients and 10,000 controls	Not reported (all ≥65 years)	31 cases; 23 controls	Nonaspirin, supply of NSAIDs included index date	An admission SCr ≥180 μmol/L (2 mg/dl) and ≥20% increase from baseline or ≥20% decline during hospitalization	1.58 (1.34,1.86); Crude OR for CKD population 1.80 (1.30, 2.50)
Schneider 2006 [37]	N-CC; Quebec universal health care program database; Canada	>65 years, filled ≥1 NSAID prescription during 01Jan1999-30June2002, NSAID prescription free ≥1 year before cohort entry	Only use aspirin, renal replacement therapy, renal transplantation, 2 NSAIDs at cohort entry	121,722 new NSAID users	78.1 cases; 78.0 controls	46.1 cases; 32.3 controls	Dispensed NSAID 1–30 days preceding the index date with no previous prescription	ICD9 584, 586	2.05 (1.61,2.60); Crude OR for CKD population 1.13 (0.79, 1.62)
Lafrance 2009 [35]	N-CC; Department of Veterans Affairs (VA) health care system; US	≥1 NSAID prescription during 01Oct2000-30Sept2006, NSAID prescription free 2 years before cohort entry	History of renal transplantation, maintenance dialysis, or AKI before cohort entry	1,432,781 new NSAID users	63 (half >65 years)	97	Single NSAID dispensed day + 30 days tolerance period with no previous prescription	Hospitalisation with AKI, AKIN definition	1.82 (1.68,1.98); Crude OR for CKD population 1.36 (1.30, 1.42)
Henry 1997 [33]	Matched CC; John Hunter Hospital and Newcastle Master Hospital; Australia	Admitted to study hospitals identified by hospital database	Unfit for interview	164 cases and 189 controls	76.6 cases; 75.1 controls	55.5 cases; 55.0 controls	Any NSAID use in past month (excluding prophylactic aspirin)	Admitted to hospitals with SCr ≥0.15 mmol/L	1.80 (0.97,3.40); Crude OR for CKD population 5.25 (1.06,26.07)

^a^CC, case-control; N-CC, nested case-control; MEMO, Medicines Monitoring Unit’s record-linkage database; ICD9, International Classification of Disease version 9; AKIN, Acute Kidney Injury Network; ACEI, angiotensin-converting-enzyme inhibitor; GPRD, General Practice Research Database; SCr, serum creatinine; tNSAID, traditional NSAID; BUN, blood urea nitrogen; CRF, chronic renal failure

^b^Definition chosen by review authors

^c^Age median (rather than mean)

^d^There are hospital controls which were ignored

**Table 3:** Confounders that the included studies adjusted for

First Author, Year	Confounders adjusted in general population
Bouvy 2003 [8]	Age and gender, prior hospital admissions for congestive heart failure, diabetes and for concomitant use of diuretics, low-dose aspirin, antibiotics, paracetamol (acetaminophen), epoetin, corticosteroids, opioids, digoxin, antigout drugs and duration of use of ACE inhibitor
Huerta 2005 [34]	Sex, age, calendar year, body mass index, HF, hypertension, diabetes, antihypertensive use, oral steroid use, NSAID use, and consultant visits and hospitalizations in the previous year
Leonard 2012 [6]	Hospitalized in prior 30 days, ever past anemia, ever past coronary disease, ever past heart failure/cardiomyopathy, ever past disorders of stomach function, ever past arthropathies and related disorders, ever past pain, ever past gastrointestinal drug use, ever past cardiovascular system drug use, ever past central nervous system drug use, ever past infection-treating drug use, ever past endocrine system drug use, ever past nutrition and blood drug use, ever past musculoskeletal and joint disease drug use, frusemide use in the prior 28 days, and kidney sensitizer drug exposure in the prior 180 days
Murray 1990 [9]	Age, gender, race, coronary artery disease, baseline systolic blood pressure, diuretic use,
Perez Gutthann 1996 [36]	Age, sex, calendar year, cardiovascular risk indicator, recent hospitalization for disorders renal, exposure to NSAIDs, prescription ASA, nephrotoxic drugs
Evans 1995 [31]	Age, gender, could not find information in other covariates
Griffin 2000 [32]	Age (65–74, 75–84, > = 85), gender, ethnicity, nursing home resident, recent hospitalization (within 30 days, 31–365 days, none in the past year), concomitant use of loop diuretic, thiazide, ACE inhibitor, and antibiotics (within 30 days), prescription for allopurinol, cyclosporin, gold, sulfinpyrazone, or penidllamine, first prescription for cimetidine in the past 60 days, or procedure code Indicating intravenous radio contrast within the past 30 days
Schneider 2006 [37]	Age, gender, comorbidity (Hypertension, Diabetes, Heart failure, Cardiovascular disease, Atherosclerosis, Hyperlipidemia, Respiratory disease, Gastrointestinal ulcer disease, Chronic renal failure, Acute renal failure, Renal disease, Renovascular disease, Renal infection, Conditions secondary to renal impairment, Renal manifestation of systemic diseases, Systemic disease and malignancy relevant to renal function), drug use (Oral anticoagulants, Oral corticosteroids, Psychotropic drugs, Thyroid drugs. Current use of aspirin, Use of nephrotoxic drugs, Exposure to contrast media), comorbidity measures (No. of different drugs, Chronic disease score, Charlson index, Health care utilization (>12 physician visits, > = 1 nephrologist visits >1 hospitalization))
Lafrance 2009 [35]	Age, gender, race, concurrent disease (Arrhythmia, Chronic kidney disease, Cardiovascular disease, Cancer, Chronic liver disease, Chronic pulmonary disease, Congestive heart failure, Diabetes, Hyperlipidemia, Hypertension, Osteoarthritis, Rheumatoid arthritis, Peptic ulcer/ GERD, PVD, Valvular disease), hospitalization (last 30 days, previous year), drug use (ACEi or ARBs, Beta-blockers, Diuretics, Oral anticoagulants, Platelet aggregation drugs, Nephrotoxic drugs, Corticosteroids, Radio contrast exposure), laboratory (Serum albumin)
Henry 1997 [33]	Age, history of gout, heart disease and renal disease

### Association between NSAID exposure and AKI in the general population

**Fig. 2:**
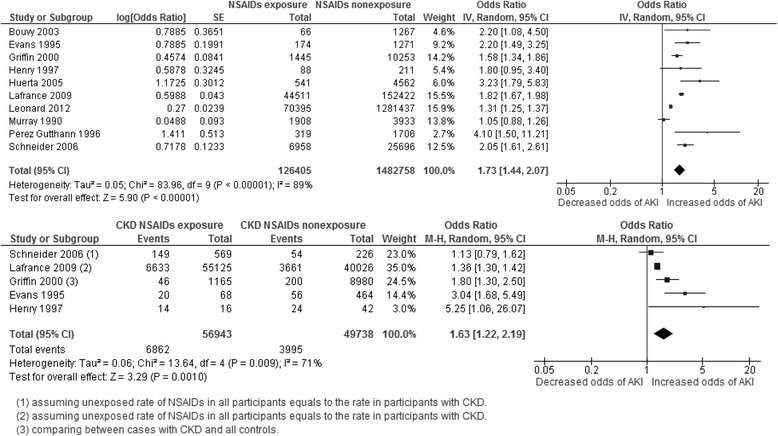
Pooled odds ratio and 95% confidence intervals for AKI in general population and people with CKD using NSAID vs not using

Ten studies that included a total of 1,609,163 participants were used to evaluate AKI risk among current NSAID users in the general population (Fig. 2). The adjusted OR for AKI was increased relative to non-users and between individual studies ranged from 1.05 to 4.10, with eight of the ten studies showing a statistically significant association between NSAID exposure and AKI. Meta-analysis of adjusted odds ratios estimated the pooled OR to be 1.73 (95%CI 1.44–2.07). Heterogeneity was substantial (I^2^ = 89%, *P* < 0.001), suggesting that the pooled estimate should be interpreted with caution.

### Association between NSAID exposure and AKI in people with CKD

Two case-control and three nested-case-control studies which included 106,681 people with CKD reported data that could be used to evaluate AKI risk by current NSAID exposure. The crude OR for AKI in NSAID users compared with nonusers ranged from 1.12 to 5.25 and was >1 and statistically significant in 4 of 5 studies (Fig. 2). The pooled crude OR was 1.63 (95% CI 1.22–2.19) and I^2^ statistic was 71% (*P* = 0.009). We noted weaker associations with AKI in larger studies with more precise estimates of risk, with the two studies reporting the largest risks being older, smaller and less precisely estimated.

### Subgroup analyses

**Fig. 3:**
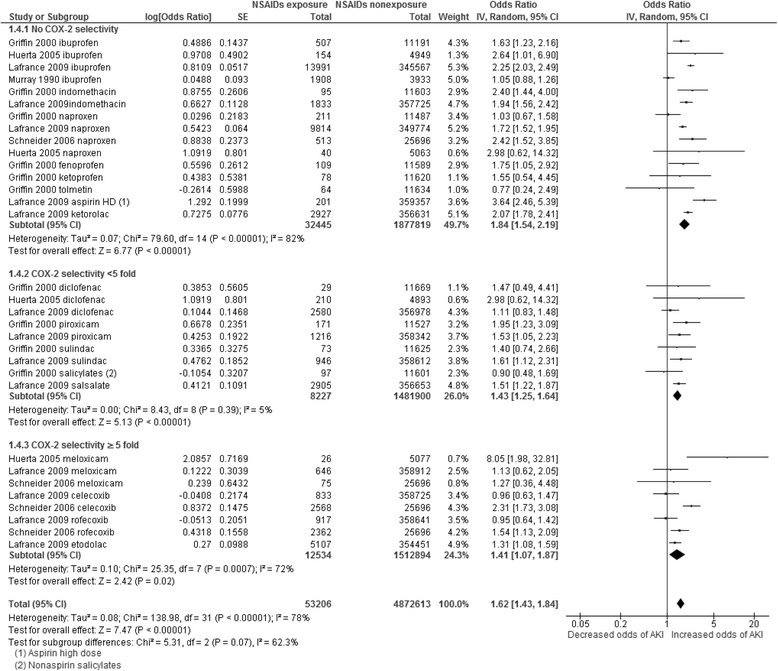
Pooled odds ratio and 95% confidence interval for AKI in general population using NSAIDs with different COX-2 selectivity vs not using

**Fig. 4:**
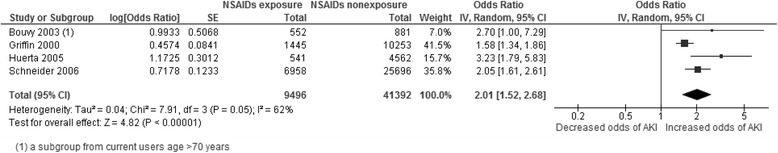
Pooled odds ratio and 95% confidence interval for AKI in elderly people using NSAIDs vs not using

**Fig. 5:**
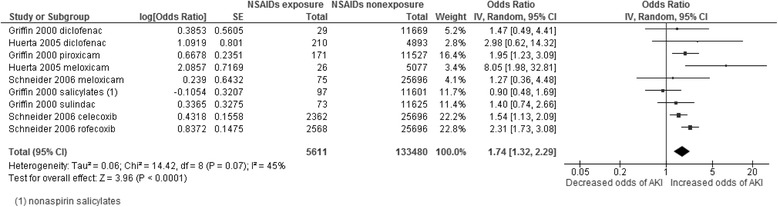
Pooled odds ratio and 95% confidence interval for AKI in elderly people using NSAIDs with COX-2 selectivity vs not using

To explore heterogeneity, we examined association with AKI in older users of NSAIDs (age > 50 years), stratified by COX-2 selectivity of the NSAIDs exposed to [38] (none, <5-fold and ≥5-fold), and in older patients with exposure to COX-2 selective NSAIDs (Figs. 3, 4 and 5). Statistical heterogeneity remained in subgroup analyses but it was modestly reduced, suggesting that subgroup analyses provided more confidence in the pooled estimates, but interpretation of pooled estimates should still be cautious.

The pooled results of NSAIDs with different COX-2 selectivity for AKI are shown in Fig. 3. Similar to the general results, there were increased associations between AKI and exposure to NSAIDs with different COX-2 selectivity (no COX-2 selectivity 1.84, 95%CI 1.54–2.19, COX-2 selectivity <5-fold 1.43, 95%CI 1.25–1.64 and COX-2 selectivity ≥5-fold 1.41, 95%CI 1.07–1.87). There was a non-statistically significant trend that the higher the COX-2 selectivity NSAIDs was, the lower the increased odds of AKI (subgroup difference test χ^2^ = 5.31, df = 2, *P* = 0.07).

Older people (>50 years) using NSAIDs had a somewhat higher odds of AKI associated with NSAID exposure than the general population (2.01, 95%CI 1.52–2.68; Fig. 4), although the confidence intervals overlap, and there was again significant heterogeneity (I^2^ = 62%). In older people who were using NSAIDs with COX-2 selectivity, the odds ratio was 1.73 (95%CI 1.32–2.29; Fig. 5) which is similar to the general population. Heterogeneity was moderate (45%) in this subgroup.

## Discussion

The results of the meta-analysis showed that current exposure to NSAIDs was associated with an approximately 1.5-fold increase in the odds of developing AKI in the general population and in people with CKD. Since AKI is a rare NSAIDs associated adverse outcome, odds ratios will approximate to relative risks. There was considerable heterogeneity between studies, particularly in the general population group and so the pooled estimates should be interpreted with caution. The limited numbers of studies eligible for inclusion precluded meta-regression, so subgroup analyses were conducted in order to try to explore and explain heterogeneity. Results were consistent with and similar to the main findings. Older people who were prescribed NSAIDs had a somewhat higher (2-fold) risk of developing AKI, but there was no strong evidence that greater COX-2 selectivity was associated with lower AKI risk. NSAIDs with high COX-2 selectivity (≥5-fold) had a lower association with AKI than NSAIDs with COX-2 selectivity <5-fold, and heterogeneity in the subgroups was reduced compared to the overall results consistent with some of the heterogeneity being due to differences in the age of the population studies and the type of NSAIDs examined. Five studies included individual NSAID usage in their analyses in which only Lafrance and Schneider compared dose effect in Rofecoxib, Celecoxib, Naproxen and Meloxicam (Lafrance only) [9, 32, 34, 35, 37]. Dose response cannot be easily stratified as higher dosage will be associated with a higher risk of effect compared to lower dosage but the exposure window is not under control. However, other differences in population and in AKI definition were substantial and likely accounts for much of the observed heterogeneity. Overall, all analyses showed a statistically significant, modestly increased risk of AKI from exposure to NSAIDs, and the magnitude of the increased risk of AKI was rather similar among all sub-groups with mostly overlapping confidence intervals.

The study strengths include careful study selection and the use of a structured quality assessment tool to ensure that only high quality studies were included [21]. The observed associations were consistent across subgroups, but the study has several limitations. As with all systematic reviews, the findings depend on the quality of the included studies. We chose to review and meta-analyse observational studies because an initial literature search identified that trials of NSAIDs rarely report renal outcomes (the focus of this study) and exclude people with CKD (a key topic of interest) and other comorbidities as well as older people and minority groups [18, 39]. However, observational studies are vulnerable to residual confounding by measured and unmeasured variables. An example is confounding by indication, which in this context is likely to occur if prescribers avoid NSAIDs in people they perceive to be at higher risk of NSAID toxicity including AKI, which would lead to an underestimation of AKI risk if present. There were also large differences between studies in the population examined and the way in which AKI was measured, both of which likely contributed to the observed moderate to large heterogeneity between studies. It is also important to recognise that the estimate of the risk of NSAIDs in the general population is adjusted for potential confounders, but the estimate in people with CKD is not because only one study reported an adjusted estimate [33]. Other limitations include that we only included studies published in English, that there were a relatively small number of studies suitable for inclusion which made meta-regression to explore heterogeneity unfeasible, and that the rate of concomitant use of OTC NSAID use could not be assessed in the populations studied. Seven of the included studies [6, 8, 31, 32, 34, 35, 37] addressed that OCT NSAID use may have caused exposure misclassification. But all of the studies believed that due to reasons such as financial incentives, the proportion of OCT NSAID users is expected to be small and nondifferential with respect to the NSAID categories the populations studied. Consequently, it would bias the results toward the null. As most of the studies confirmed eligible cases then selected controls according to a certain proportion, non-response rate was not given in the majority of the included studies. Since cases and controls of these studies were derived from the same databases, were examined for the same exposure and followed up in the same way, then non-response rate is not considered to be a problem and therefore it is unlikely there would be missing data bias. We were unable to access publication bias because of the extensive statistical heterogeneity. For many of the methodological qualities assessed there was an unclear risk of bias as studies did not provide explicit detail to make an informed judgement.

To our knowledge, this study is the first meta-analysis to examine associations between NSAID exposure and AKI in the general community-dwelling population and people with CKD. A previous systematic review which was conducted in 2014 included five observational studies and reported risk of AKI by individual NSAID exposure, finding a statistically significant elevated AKI risk among most of the traditional NSAIDs but did not achieve a statistical significance for COX-2 inhibitors or traditional NSAIDs with higher COX-2 selectivity (meloxicam and diclofenac) [40]. Our study used a more comprehensive search and included additional studies, and found a similar estimate of pooled risk but statistically significantly increased risk irrespective of COX-2 selectivity. Another systematic review specifically focused on myocardial, vascular and renal risks of COX-2-selective meloxicam allowed a broad definition of renal outcomes and it did not find a significantly increased renal risk [41]. A third systematic review examined the risk of CKD progression associated with NSAID use, finding that high (but not standard) dose NSAID use was associated with an increased risk of CKD progression [42].

## Conclusion

AKI is an increasingly common global problem causing significant morbidity and mortality and with large resource implications. Exposure to NSAIDS and other nephrotoxic drugs is an important cause of AKI, but the risk of these exposures is modified by susceptibilities such as increasing age and the presence of CKD [43]. This study found that the odds of developing AKI increased by over 50% in people who were exposed to NSAIDs in the general population and in people with CKD, and in older people the odds of developing AKI doubled. However, the absolute risk of developing AKI also depends on the baseline risk of AKI in the population exposed, which none of the included studies reported. Future studies should use internationally agreed definitions of AKI [44] and estimate the absolute risk of AKI in different populations including older people and people with CKD to better inform clinical decision making. There is evidence that feedback and more complex interventions to reduce NSAID prescribing in people at high risk of renal adverse effects are effective [45, 46], and clinicians should seek to minimise NSAID exposure in people particularly susceptible to AKI due to age, CKD or because of the co-prescription of other nephrotoxic drugs [43, 47].

## Additional files

Additional file 1: Complete search strategy. The complete search strategy for the systematic review for both Medline and Embase. (DOCX 22 kb)

Additional file 2: MOOSE Checklist. Essential items to report in meta-analysis of observational studies in epidemiology. (DOCX 26 kb)

Additional file 3: Detailed reasons for excluding full text. Detailed reasons for 20 excluded papers at full text papers reviewing stage with reference. (DOCX 26 kb)

## Abbreviations

ACE: Inhibitors angiotensin-converting-enzyme inhibitor
ADEs: Adverse drug events
AIN: Interstitial nephritis
AKI: Acute kidney injury
ASA: Acetylsalicylic acid
CI: Confidence interval
CKD: Chronic kidney disease
CrCl: Creatinine clearance
GFR: Glomerular filtration rate
ICD: International Classification of Diseases
KDIGO: Kidney Disease Improving Global Outcomes
MOOSE: Meta-analysis of observational studies in epidemiology
NSAIDs: Non-steroidal anti-inflammatory drugs
OR: Odds ratio
OTC: Over the counter
RR: Relative risk
SCr: Serum creatinine
UK: United Kingdom
US: United States
