# Supplementary material for: Non-steroidal anti-inflammatory drug induced acute kidney injury in the community dwelling general population and people with chronic kidney disease: systematic review and meta-analysis
Source: BMC Nephrol. 2017 Aug 1;18:256. doi: 10.1186/s12882-017-0673-8 (PMC5540416; doi:10.1186/s12882-017-0673-8)
Supplement: Supplementary file 1 — Complete search strategy. The complete search strategy for the systematic review for both Medline and Embase. (DOCX 22 kb) [file 12882_2017_673_MOESM1_ESM.docx]

**Additional File 1. Complete search strategy**

**Medline 1946 to 2016 June 21**

**Embase 1974 to 2016 June 21**

1. exp Anti-Inflammatory Agents, Non-Steroidal/
2. (non-steroidal anti-inflammatory adj (agent* or drug*)).tw.
3. (nonsteroidal anti-inflammatory adj (agent* or drug*)).tw.
4. (non-steroidal antiinflammatory adj (agent* or drug*)).tw.
5. (nonsteroidal antiinflammatory adj (agent* or drug*)).tw.
6. (Anti-Inflammator$ or (Anti adj Inflammator$) or AntiInflammator$).tw.
7. NSAID*.tw.
8. Cyclooxygenase 2 Inhibitors/
9. cox 2 inhibitor$.tw.
10. cyclooxygenase 2 inhibitor$.tw.
11. cyclo-oxygenase-2 inhibitor$.tw.
12. coxib$.tw.
13. apazone.tw.
14. aspirin.tw.
15. clonixin.tw.
16. diclofenac.tw.
17. diflunisal.tw.
18. epirizole.tw.
19. fenoprofen.tw.
20. feprazone.tw.
21. flurbiprofen.tw.
22. ibuprofen.tw.
23. indomethacin.tw.
24. ketoprofen.tw.
25. ketorolac.tw.
26. meclofenamic acid.tw.
27. mefenamic acid.tw.
28. naproxen.tw.
29. niflumic acid.tw.
30. phenylbutazone.tw.
31. piroxicam.tw.
32. alicylate$.tw
33. Sodium salicylate.tw.
34. sulindac.tw.
35. tolmetin.tw.
36. celecoxib.tw.
37. etodolac.tw.
38. tenoxicam.tw.
39. parecoxib.tw.
40. rofecoxib.tw.
41. meloxicam.tw.
42. valdecoxib.tw.
43. (movalis or mobec or mobic or movicox or mobicox or parocin or uticox or etoricoxib or arcoxia or celecoxib or celebrex).tw.
44. Acetylsalicylic acid.tw.
45. acephen.tw.
46. Ampyrone.tw.
47. Amynopirin.tw.
48. Antipyrine.tw.
49. Bufexamac.tw.
50. Clofazimine.tw.
51. Curcumin.tw.
52. Dipyrone.tw.
53. Fenbufen.tw.
54. Floctafenine.tw.
55. Lederfen.tw.
56. Mesalamine.tw.
57. Niflumic Acid.tw.
58. Phenazone.tw.
59. Seractil.tw.
60. Suprofen.tw.
61. Acemetacin.tw.
62. Aceclofenac.tw.
63. Clofenamic acid.tw.
64. Dexketoprofen.tw.
65. Dexibuprofen.tw.
66. Etoricoxib.tw.
67. Lornoxicam.tw.
68. Nabumetone.tw.
69. Nimesulide.tw.
70. Seractil.tw.
71. Suprofen.tw.
72. Tiaprofenic acid.tw.
73. or/1-72
74. Kidney/
75. Renal Insufficiency/
76. exp Acute Kidney Injury/
77. Creatinine/
78. Kidney Function Tests/
79. (kidney* or renal).tw.
80. creatinine.tw.
81. (nephrotoxic* or nephrotoxin).tw.
82. azot?emia.tw.
83. glomerular filtration rate.tw.
84. (gfr or egfr).tw.
85. kidney diseases/ or exp renal insufficiency/
86. exp Nephritis/
87. Nephrotic Syndrome/
88. exp Nephrolithiasis/
89. exp kidney/ and exp “wounds and injuries”/

PUT exp kidney/ and (exp wounds/ or exp injuries/) IN OVID SEARCH

1. ((kidney$ or renal$ or nephr$) adj5 (disease$ or syndrome or impair$ or insuffic$ or function$ or dysfunction$ or disorder$ or thrombosis or stenosis or injur$ or damage$ or fibrosis or aneurysm$or necrosis$ or failure)).tw.
2. Nephriti$.tw.
3. glomerulonephritis.tw.
4. glomerulosclerosis.tw.
5. nephrolithiasis.tw.
6. or/74-94
7. and/73,95
8. Epidemiologic studies/
9. Exp case control studies/
10. Exp cohort studies/
11. Case control.tw.
12. (cohort adj (study or studies)).tw.
13. Cohort analy$.tw.
14. (Follow up adj (study or studies)).tw.
15. (observational adj (study or studies)).tw.
16. Longitudinal.tw.
17. Retrospective.tw.
18. Cross sectional.tw.
19. Cross-sectional studies/
20. or/97-108
21. and/96,109
22. or/97-106
23. and/96,111
