# Supplementary material for: Non-steroidal anti-inflammatory drug induced acute kidney injury in the community dwelling general population and people with chronic kidney disease: systematic review and meta-analysis
Source: BMC Nephrol. 2017 Aug 1;18:256. doi: 10.1186/s12882-017-0673-8 (PMC5540416; doi:10.1186/s12882-017-0673-8)
Supplement: Supplementary file 2 — MOOSE Checklist. Essential items to report in meta-analysis of observational studies in epidemiology. (DOCX 26 kb) [file 12882_2017_673_MOESM2_ESM.docx]

**Additional File 3.** MOOSE Checklist

| **Checklist Item** | **Item #** | **Reported on page #** |
| --- | --- | --- |
| **Title** |  |  |
| Identify the study as a systematic review, meta-analysis, or both | 1 | 1 |
| **Abstract** |  |  |
| Provide a structured summary including, as applicable: background; objectives; data sources; study eligibility criteria, participants, and interventions; study appraisal and synthesis methods; results; limitations; conclusions and implications of key findings; systematic review registration number | 2 | 2 |
| **Introduction** |  |  |
| Problem definition | 3 | 3 |
| Hypothesis statement | 4 | 3-4 |
| Description of Study population, type of exposure or intervention used, comparisons, study outcomes and study design (PICOS) | 5 | 4 |
| **Method** |  |  |
| Qualifications of searchers (eg librarians and investigators) | 6 | 1, 19 |
| Search strategy, including time period used in the synthesis and key words | 7 | 5 p1,  Additional File 1 |
| Effort to include all available studies, including contact with authors | 8 | 5 p1-2 |
| Databases and registries searched | 9 | 5 p1 |
| Search software used, name and version, including special features used (eg explosion) | 10 | 5 p1 |
| Use of hand searching (eg reference lists of obtained articles) | 11 | 5 p1 |
| List of citations located and those excluded, including justification | 12 | Figure 1,  Additional File 2 |
| Method of addressing articles published in languages other than English | 13 | 5 p1 |
| Method of handling abstracts and unpublished studies | 14 | 5 p2 |
| Description of any contact with authors | 15 | 5 p2 |
| Description of relevance or appropriateness of studies assembled for assessing the hypothesis to be tested | 16 | 6 |
| Rationale for the selection and coding of data (eg sound clinical principles or convenience) | 17 | 5 p3 |
| Documentation of how data were classified and coded (eg multiple raters, blinding and interrater reliability) | 18 | 5-6 |
| Assessment of confounding (eg comparability of cases and controls in studies where appropriate) | 19 | 6 |
| Assessment of study quality, including blinding of quality assessors, stratification or regression on possible predictors of study results | 20 | 6-7 |
| Assessment of heterogeneity | 21 | 7 |
| Description of statistical methods (eg complete description of fixed or random effects models, justification of whether the chosen models account for predictors of study results, dose-response models, or cumulative meta-analysis) in sufficient detail to be replicated | 22 | 7 |
| Provision of appropriate tables and graphics | 23 | 23-27 |
| **Results** |  |  |
| Graphic summarizing individual study estimates and overall estimate | 24 | Figure 2 |
| Table giving descriptive information for each study included | 25 | 23-24 |
| Results of sensitivity testing (eg subgroup analysis) | 26 | 11-12 |
| Indication of statistical uncertainty of findings | 27 | 11-12 |
| **Discussion** |  |  |
| Strengths and weaknesses | 28 | 14-15 |
| Quantitative assessment of bias (eg publication bias) | 29 | 15 |
| Justification for exclusion (eg exclusion of non-English language citations) | 30 | 14 |
| Assessment of quality of included studies | 31 | 14 |
| Consideration of alternative explanations for observed results | 32 | 13-14 |
| Generalization of the conclusions (eg appropriate for the data presented and within the domain of the literature review) | 33 | 16 |
| Guidelines for future research | 34 | 16 |
| Disclosure of funding source | 35 | 18 |

*Transcribed and modified from the original paper by Xinyu Zhang. February 2015.

** ‘p’ in ‘Reported on page #’ column means paragraph

From: [Donna F. Stroup](http://jama.ama-assn.org/search?author1=Donna+F.+Stroup&sortspec=date&submit=Submit), PhD, MSc; [Jesse A. Berlin](http://jama.ama-assn.org/search?author1=Jesse+A.+Berlin&sortspec=date&submit=Submit), ScD; [Sally C. Morton](http://jama.ama-assn.org/search?author1=Sally+C.+Morton&sortspec=date&submit=Submit), PhD; [Ingram Olkin](http://jama.ama-assn.org/search?author1=Ingram+Olkin&sortspec=date&submit=Submit), PhD; [G. David Williamson](http://jama.ama-assn.org/search?author1=G.+David+Williamson&sortspec=date&submit=Submit), PhD; [Drummond Rennie](http://jama.ama-assn.org/search?author1=Drummond+Rennie&sortspec=date&submit=Submit), MD; [David Moher](http://jama.ama-assn.org/search?author1=David+Moher&sortspec=date&submit=Submit), MSc; [Betsy J. Becker](http://jama.ama-assn.org/search?author1=Betsy+J.+Becker&sortspec=date&submit=Submit), PhD; [Theresa Ann Sipe](http://jama.ama-assn.org/search?author1=Theresa+Ann+Sipe&sortspec=date&submit=Submit), PhD; [Stephen B. Thacker](http://jama.ama-assn.org/search?author1=Stephen+B.+Thacker&sortspec=date&submit=Submit), MD, MSc; for the Meta-analysis Of Observational Studies in Epidemiology (MOOSE) Group. **Meta-analysis of Observational Studies in Epidemiology. A Proposal for Reporting** JAMA. 2000;283(15):2008-2012. doi: 10.1001/jama.283.15.2008
