# Supplementary material for: Non-steroidal anti-inflammatory drug induced acute kidney injury in the community dwelling general population and people with chronic kidney disease: systematic review and meta-analysis
Source: BMC Nephrol. 2017 Aug 1;18:256. doi: 10.1186/s12882-017-0673-8 (PMC5540416; doi:10.1186/s12882-017-0673-8)
Supplement: Supplementary file 3 — Detailed reasons for excluding full text. Detailed reasons for 20 excluded papers at full text papers reviewing stage with reference. (DOCX 26 kb) [file 12882_2017_673_MOESM3_ESM.docx]

**Additional File 2. Detailed reasons for excluding full text**

30 full text papers reviewed, 10 included in meta-analysis, 20 excluded

Detailed reasons for study exclusion are presented below: two studies were published conference abstracts (1,2); six studies did not report AKI data in a usable format (3-8); three studies used progression of CKD as outcome (9-11); one study comment in the text about NSAID risks of renal impairment in people with pre-existing CKD with no actual data (12); two studies provided incidence rate ratio that could not be synthesized with odds ratio (13), the other only provided rate ratio with individual NSAID (14); two studies exposure was a combination of drugs rather than NSAIDs (15, 16); one study specifically excluded CKD (17); one study used duplicate population as one of the included studies (18); the last two excluded studies had no comparison group (19, 20).

**Not original investigation=2**

***Kuo HW, 2010 (Nephrology)***

Published conference abstract

***Yarger S, 2011***

Published conference abstract

**No outcomes of interest=9**

***Agodoa LY, 2008***

No information about AKI, Cross sectional study of ‘ever habitual analgesic use’ and ‘current renal function’ in forms of albuminuria and reduced eGFR

***Allred J, 1989***

Not mentioned AKI, Cross sectional case control study of current NSAID user vs not current NSAID user, examining associations with current renal function comparing mean and standard association (with 95% CI) of urea, creatinine and potassium.

***Atkinson M, 1991***

No information about AKI, no patient had to be withdrawn from the study because of an abnormal renal test result; Post hoc analysis of trial data

***Calvo-Alen J, 1994***

No information about AKI, compared serum creatinine mean and SD with student’s t test between rheumatic patients and healthy controls

***Field T, 1999***

No information about AKI, compared serum creatinine mean and SD between NSAID users and non NSAID users

***Lafrance JP, 2012***

No information about AKI, outcome hyperkalaemia not AKI

***Gooch K, 2007***

Outcome is progression of CKD

***Hippisley-Cox J, 2010***

Outcome is progression of CKD

***Kuo HW, 2010 (Pharmacoepidemiology & Drug Safety)***

Outcome is progression of CKD

**No usable data=3**

***Dieppe P, 2004***

NSAID risks of renal impairment in people with pre-existing CKD is a comment in the text with no numbers; and even the data they do report is pretty skimpy reflecting that this is an ‘analysis’ rather than a ‘research’ paper; Conclude pitfalls in RCT including criteria

***Pratt N, 2010***

Cohort study, Incident rate ratio cannot be synthesis with OR, reference subgroup can be used. Other 2 cohorts are diabetes patients and ACEI users.

***Winkelmayer WC, 2008***

Rate ratio cannot be synthesised. No overall result, separate NSAID RR might be used.

**No comparison group=2**

***Jayasutha J, 2012***

***de Vries F, 2010***

**Wrong population=4**

***Lapi F, 2013***

Focus is nephrotoxic effect of drug-drug interaction (triple whammy of ACE inhibitor/ Angiotension Receptor Blocker + diuretic + NSAID)

***Dreischulte T, 2015***

Focus is nephrotoxic effect of drug-drug interaction (triple whammy of ACE inhibitor/ Angiotension Receptor Blocker + diuretic + NSAID)

***Chou C, 2016***

Analysis excludes people with CKD so ‘general population’ is not comparable to other studies

***Guess HA, 1985***

Reports a very similar analysis of the same database as the study by Gutthann P (1996). Included the study by Gutthann P as it is newer and has better study quality in Newcastle-Ottawa scale.

***<100 participants=2**

****Jayasutha J, 2012*** 40 total participants

****Allred J, 1989*** 54 total participants

*other reasons to exclude the studies apart from the reasons mentioned above

**20 excluded studies references:**

1. Kuo HW, Yang CY, Tsai SS, Liu YC. The renal effects of conventional and selective nonsteroidal anti-inflammatory drugs in chronic kidney disease patients. Nephrology. 2010 June;15:84-5. PubMed PMID: 70467551.
2. Yarger S, Nwokeji E, Trice S, Chao S, Devine J, Potyk R, et al. Cumulative exposure to nonsteroidal anti-inflammatory drugs (nsaids) and the progression of chronic kidney disease (ckd). Value in Health. 2011 May;14 (3):A74-A5. PubMed PMID: 70490838.
3. Agodoa LY, Francis ME, Eggers PW. Association of analgesic use with prevalence of albuminuria and reduced GFR in US adults. American Journal of Kidney Diseases. 2008;51(4):573-83. PubMed PMID: 18371533.
4. Allred J, Wong W, Kafetz K. Elderly people taking non-steroidal anti-inflammatory drugs are unlikely to have excess renal impairment. Postgraduate Medical Journal. 1989;65(768):735-7. PubMed PMID: 2616399. Pubmed Central PMCID: PMC2429830.
5. Atkinson M, Basch C, Brett L. Long-term renal and hepatic tolerability of naproxen: A review of effects in young and elderly patients. Clinical Therapeutics. 1991;13(SUPPL. A):44-50. PubMed PMID: 1991251246.
6. Calvo-Alen J, De Cos MA, Rodriguez-Valverde V, Escallada R, Florez J, Arias M. Subclinical renal toxicity in rheumatic patients receiving longterm treatment with nonsteroidal antiinflammatory drugs. Journal of Rheumatology. 1994;21(9):1742-7. PubMed PMID: 7799360.
7. Field TS, Gurwitz JH, Glynn RJ, Salive ME, Gaziano JM, Taylor JO, et al. The renal effects of nonsteroidal anti-inflammatory drugs in older people: findings from the Established Populations for Epidemiologic Studies of the Elderly. Journal of the American Geriatrics Society. 1999;47(5):507-11. PubMed PMID: 10323640.
8. Lafrance JP, Miller DR. Dispensed selective and nonselective nonsteroidal anti-inflammatory drugs and the risk of moderate to severe hyperkalemia: a nested case-control study. American Journal of Kidney Diseases. 2012;60(1):82-9. PubMed PMID: 22503390.
9. Gooch K, Culleton BF, Manns BJ, Zhang J, Alfonso H, Tonelli M, et al. NSAID use and progression of chronic kidney disease. American Journal of Medicine. 2007;120(3):280.e1-7. PubMed PMID: 17349452.
10. Hippisley-Cox J, Coupland C. Predicting the risk of chronic Kidney Disease in men and women in England and Wales: prospective derivation and external validation of the QKidney Scores. BMC Family Practice. 2010;11:49. PubMed PMID: 20565929. Pubmed Central PMCID: PMC2905345.
11. Kuo HW, Tsai SS, Tiao MM, Liu YC, Lee IM, Yang CY. Analgesic use and the risk for progression of chronic kidney disease. Pharmacoepidemiology & Drug Safety. 2010;19(7):745-51. PubMed PMID: 20582905.
12. Dieppe P, Bartlett C, Davey P, Doyal L, Ebrahim S. Balancing benefits and harms: The example of non-steroidal anti-inflammatory drugs. British Medical Journal. 2004 03 Jul;329(7456):31-4. PubMed PMID: 2004287614.
13. Pratt N, Roughead EE, Ryan P, Gilbert AL. Differential impact of NSAIDs on rate of adverse events that require hospitalization in high-risk and general veteran populations: a retrospective cohort study. Drugs & Aging. 2010;27(1):63-71. PubMed PMID: 20030433.
14. Winkelmayer WC, Waikar SS, Mogun H, Solomon DH. Nonselective and cyclooxygenase-2-selective NSAIDs and acute kidney injury. American Journal of Medicine. 2008;121(12):1092-8. PubMed PMID: 19028206.
15. Lapi F, Azoulay L, Yin H, Nessim SJ, Suissa S. Concurrent use of diuretics, angiotensin converting enzyme inhibitors, and angiotensin receptor blockers with non-steroidal anti-inflammatory drugs and risk of acute kidney injury: nested case-control study. BMJ. 2013;346:e8525. PubMed PMID: 23299844. Pubmed Central PMCID: PMC3541472.
16. Dreischulte T, Morales DR, Bell S, Guthrie B: Combined use of nonsteroidal anti-inflammatory drugs with diuretics and/or renin-angiotensin system inhibitors in the community increases the risk of acute kidney injury. Kidney Int 2015. PMID: 25874600.
17. Chou C-I, Shih C-J, Chen Y-T, et al. Adverse Effects of Oral Nonselective and cyclooxygenase-2-Selective NSAIDs on Hospitalization for Acute Kidney Injury: A Nested Case–Control Cohort Study. Malindretos. P, ed. Medicine. 2016;95(9):e2645. Pubmed PMID: 26945352. Pubmed Central PMCID: PMC4782836.
18. Guess HA, Strand LM, Helston N, Lydick E, Berg-man U,Wolski K. Hospitalizations for renal impairment among users and non-users of non-steroidal anti-inflammatory drugs in Saskatchewan, Canada, 1983. In: Rainsford KD, Velo GP, editors. Sideeffects of anti-inflammatory drugs, part 2. Lancaster, UK: MTP; 1985. p. 367–74.
19. de Vries F, Setakis E, van Staa TP. Concomitant use of ibuprofen and paracetamol and the risk of major clinical safety outcomes. British Journal of Clinical Pharmacology. 2010;70(3):429-38. PubMed PMID: 20716244. Pubmed Central PMCID: PMC2949916.
20. Jayasutha J, Nisha MR, Bhargav dilip S, Ramasamy C. Assessment of NSAIDS induced renal diseases. International Journal of Pharmaceutical Sciences Review and Research. 2012 November/December;17(2):77-9. PubMed PMID: 2013015456.
